# Pre-acceptance study of bi-monthly Aripiprazole in clinically stable patients

**DOI:** 10.1192/j.eurpsy.2025.2098

**Published:** 2025-08-26

**Authors:** S. Sanchez-Alonso, S. Manchado, A. Loewen, L. Navio Garcia, S. Ovejero

**Affiliations:** 1 Psychiatry, University Hospital Fundación Jiménez Díaz, Madrid, Spain

## Abstract

**Introduction:**

Aripiprazole monthly (Ar1M) has been the first long-acting injectable (LAI) partial agonist antipsychotic. The benefits of long-acting injectables in terms of relapse reduction are well known.

**Objectives:**

The aim of this study is to assess the level of acceptance and the doubts presented by patients before switching to 2-monthly Aripiprazole (Ar2M).

**Methods:**

25 patients diagnosed with schizophrenia and related disorders in symptomatic remission were asked consecutively whether they would switch to the new bimonthly formulation of aripiprazole and the doubts expressed were collected.

**Results:**

The sample is composed of 25 patients (12 women and 13 men). The mean age is 52.64 years. All are being treated with Ar1M with a mean dose of 408 mg/monthly. Most of the patients present a diagnosis of affective psychosis (N=12 (42%)), 36% a non-affective psychosis (N=9) and 16% a delusional disorder (N=4). Presenting an average of 3.8 previous admissions.

Acceptance was mostly positive, with an initial acceptance rate of 76 % (N=19). Twelve percent (N=3) did not initially want the treatment. Another 12% had doubts and preferred to postpone the decision. 20% of the patients had doubts, related to possible appearance of side effects. 75% of the patients who do not want the treatment have doubts, as do the patients who prefer to wait. Of the patients who initially accepted the treatment, only 1 expressed doubts about it.

**Conclusions:**

The level of acceptance of Ar2M is very high, exceeding 75%. Of the doubts expressed about the possible change, the appearance of side effects is a matter of concern. Given the high level of acceptance, the treatment proposal is important given the wide-ranging benefits it can bring to patients. The clarification of doubts and the successive proposals of the treatment can contribute to a greater acceptance.

**Disclosure of Interest:**

None Declared

